# Supplementary material for: SARS-CoV-2 Accessory Protein ORF8 Decreases Antibody-Dependent Cellular Cytotoxicity
Source: Viruses. 2022 Jun 7;14(6):1237. doi: 10.3390/v14061237 (PMC9230529; doi:10.3390/v14061237)
Supplement: Supplementary file 1 [file viruses-14-01237-s001.zip › viruses-1682997-supplementary.pdf]

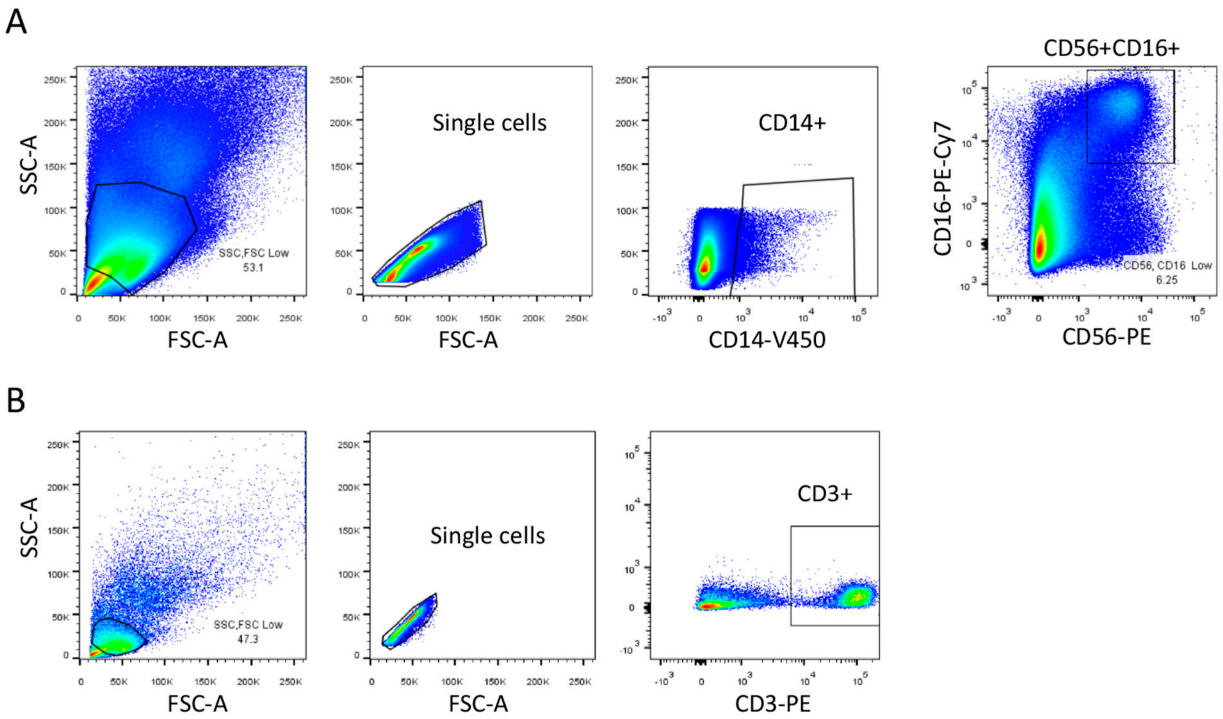

Supplementary Figure S1. ORF8 binding to PBMCs. (A) Gating strategy for CD14+ monocytes and CD56+CD16+ NK cells. Anti-CD14-V450, anti-CD56-PE and anti-CD16-PE-Cy7 were used to stain PBMCs. **(B)** Gating strategy for CD3+ T cells. PBMCs were incubated with anti-CD3-PE antibody on ice for 1 hour to label CD3+ T cells.

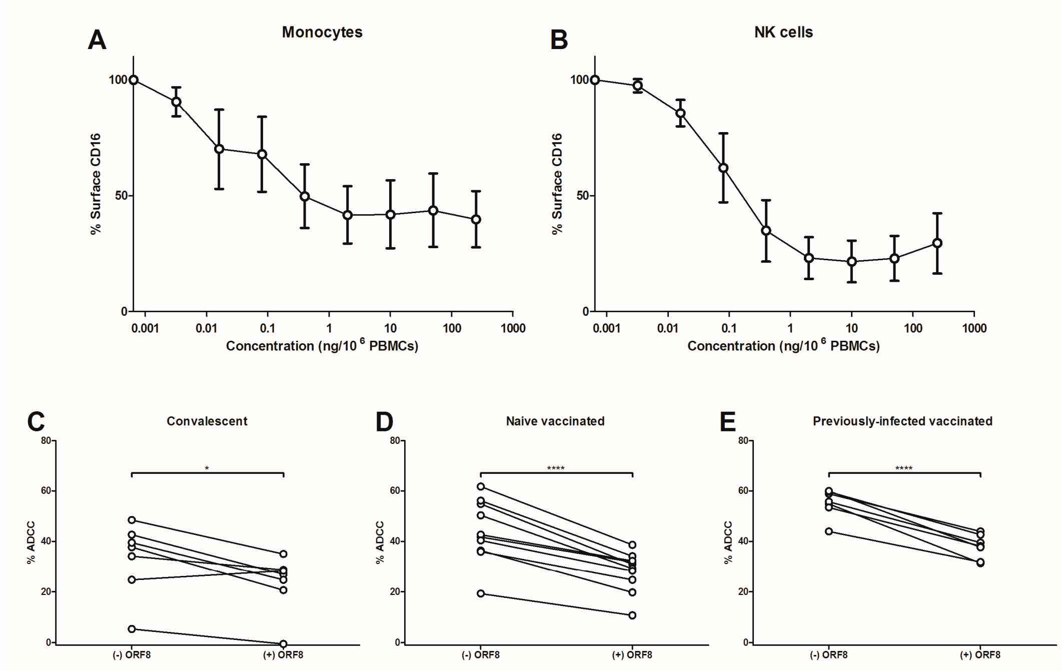

Supplementary Figure S2. Effects of recombinant ORF8 on CD16 surface level and ADCC. PBMCs from 2 different donors were thawed and incubated overnight (16 hours) with increasing concentrations of recombinant ORF8 (from 3.2 pg10^6 PBMCs to 250 ng/10^6 PBMCs). Following treatment, PBMCs were stained and flow cytometry was used to separate (**A**) Monocytes and (**B**) NK cells. (**C**) ADCC (%) mediated by plasma from 7 convalescent individuals, (**D**) 10 vaccinated individuals and (**E**) 7 previously-infected vaccinated individuals using PBMCs from a healthy donor as effector cells. The PBMCs were treated overnight (16 hours) with media from ORF8 DNA-transfected HEK293T cells (+ORF8) or with an identical volume of conditioned media collected from HEK293T cells transfected with a control plasmid (-ORF8). Statistical significance was evaluated using a parametric t-test for (**C**), (**D**) and (**E**). *, $p < 0.05$; ****, $p < 0.0001$. (**A**) and (**B**) Cell-surface CD16 levels in presence of ORF8 were normalized on cell-surface CD16 detected in absence of ORF8. Mean values ± the standard error of the mean (SEM).

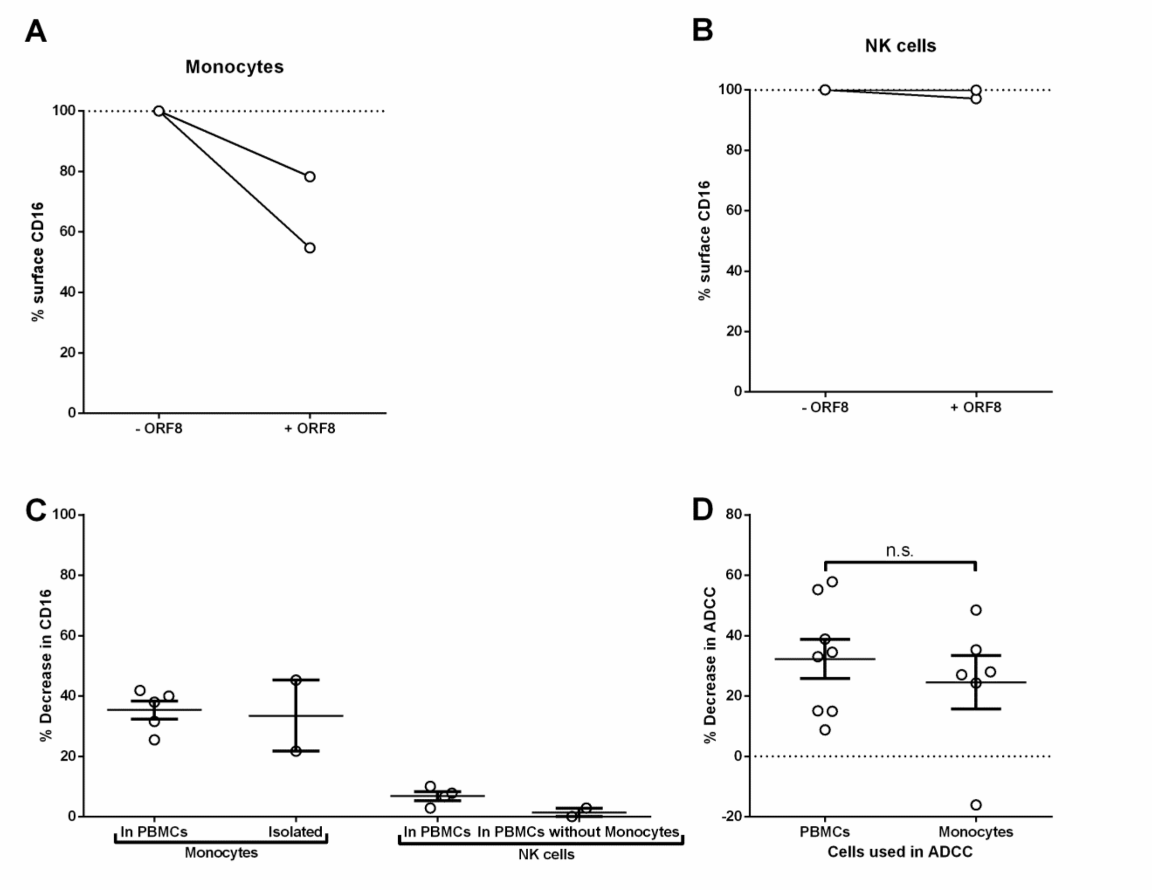

Supplementary Figure S3. ORF8 modulation of CD16 levels at the surface of purified monocytes and NK cells. (**A**) Purified monocytes from PBMCs were treated (16 hours) or not with ORF8 and cell surface levels of CD16 were measured by flow cytometry, *n*=2. (**B**) Monocytes-depleted PBMCs were treated (16 hours) or not with ORF8 and CD16 levels were measured at the surface of NK cells, *n*=2. Purified monocytes and monocytes-depleted PBMCs were treated with media from ORF8 DNA-transfected HEK293T cells (+ORF8) or with an identical volume of conditioned media collected from HEK293T cells transfected with a control plasmid (-ORF8). (**C**) Decrease of surface CD16 after ORF8 treatment on monocytes present in the PBMC population, on isolated monocytes, on NK cells in the PBMC population and on NK cells in monocytes-depleted PBMCs. (**D**) Decrease of ADCC mediated by PBMCs or purified monocytes after treatment with ORF8 for 16 hours. Statistical significance was evaluated using a non-parametric Mann-Whitney test for (**D**). n.s., not significant. (**C**) and (**D**) Mean values ± the standard error of the mean (SEM).

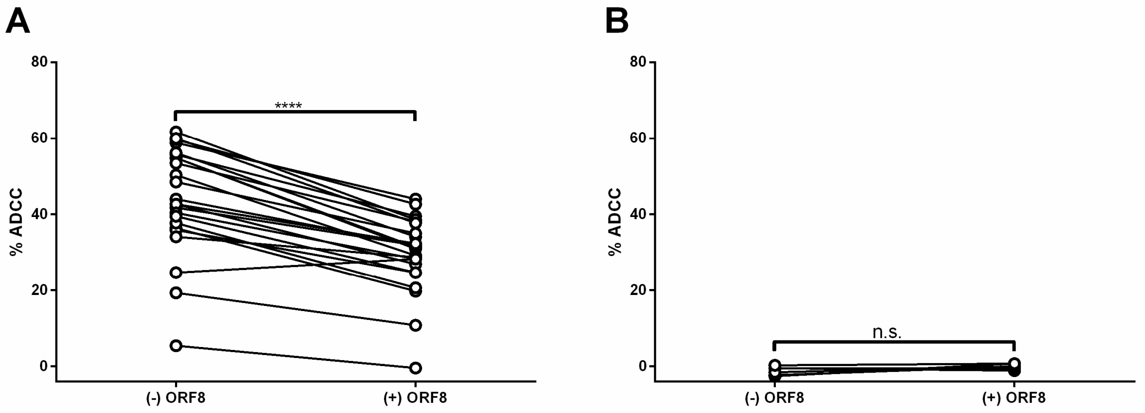

Supplementary Figure S4. Impact of ORF8 on ADCC mediated by plasma from uninfected/unvaccinated and infected and/or vaccinated individuals. ADCC mediated by plasma from infected and/or vaccinated individuals (**A**) or uninfected and unvaccinated individuals (**B**) performed in the presence (+) or absence (-) of recombinant ORF8 (0,05 µg ORF8/106 PBMCs). Data was tested for normality and a paired t-test was used to calculate significance of the data. **** : $p < 0.0001$; n.s. : non-significant ($p > 0.05$).
